# Prosper or beggar thy neighbour: Industrial policy effect of free trade zones

**DOI:** 10.1371/journal.pone.0293444

**Published:** 2023-10-30

**Authors:** Jane Du, Cheng King, Xinxiang Shi

**Affiliations:** 1 The China Institute, SOAS, University of London, London, United Kingdom; 2 Institute of Governance, Shandong University, Qingdao, China; 3 Institute of Free Trade Zones, Sun Yat-Sen University, Guangzhou, China; Cavendish University / Kyambogo University, UGANDA

## Abstract

Firms that stand to gain from the institutional framework of a free trade zone (FTZ) usually opt for locations in the FTZ region where they could expect higher returns on investment. This concentration of industries in FTZs can result in a reallocation of productivity, potentially leading to the “hollowing out” effect in existing industries, which can have a beggar-thy-neighbour effect on regional growth. Over the long term, the outcome, whether it leads to prosperity or detriment, hinges on the delicate balance between the immediate static loss associated with resource reallocation and the dynamic gains based on traditional manufacturing sector’s growth along the evolving FTZ environment.

## 1. Introduction

The nationwide success of economic zones in China has given rise to significant implications for protectionist policies, indicating a shift in policymakers’ strategies towards incorporating regional industrial policies into broader economy-wide competition policies. Unlike the trend towards political centralisation, during much of the reform period, China’s local governments enjoyed substantial autonomy in shaping economic policies, contributing to the development of many thriving regional economies.

The Chinese Communist Party’s longstanding tradition of using growth-based criteria, such as local GDP growth, to evaluate and promote officials has led to a phenomenon akin to a “promotion competition” [1–3] among Party members. Their enthusiastic pursuit of boosting regional growth effectively acts as a form of industrial policy, particularly evident in the case of economic zones. China’s success in fostering growth through economic zones underscores the significance of granting higher economic autonomy to these zones, which in turn empowers local governors and enhances their competitiveness within the Party’s “promotion competition”. This symbiotic relationship further solidifies economic zones as autonomous entities capable of generating substantial quasi-industrial-policy effects, particularly in industries aimed at boosting GDP.

This institutional autonomy of economic zones has continued to expand, most notably within the latest iteration of these zones, known as the Pilot Free Trade Zone (*hereafter* FTZ). Some of today’s FTZs are geographically located within earlier-established economic zones, such as Shenzhen and Hainan. In essence, n for some FTZs, they are categorised as a type of an economic zone within an economic zone, wielding the highest level of decision-making power among all types of zones in China. When superimposed on land and population size, the quasi-industrial-policy effect becomes especially pronounced in FTZs.

Indeed, there have been anomalous factor returns identified within existing economic zones [4, 5], which were once thought to be a natural consequence of zones’ growth momentum [6]. However, when geographic variations in factor returns are observed within the same industry, the notion of general growth becomes less convincing.

One plausible hypothesis to explain these geographical disparities is the underlying reason behind their occurrence. Some empirical studies suggest that this phenomenon arises due to the preferential policies granted to zones, which attract firms away from neighbouring areas [7], thereby exacerbating economic disparities both within and outside the zone. Consequently, the observed geographic variations in factor returns can be largely attributed to the differential subsidies and/or rent-seeking activities of local governments.

This paper draws inspiration from empirical studies conducted on FTZs [8], particularly focusing on the policy-driven industry agglomeration [9–11] and distribution of firm productivity [12, 13] in the network of economic zones [14, 15]. A key political selling point for economic zone is their role in promoting regional development by attracting specific industries to particular locations. Thus, a distinctive characteristic of Chinese economic zones lies in their *de facto* industrial policy impact. This policy-induced agglomeration primarily draws in industries that contribute significantly to GDP and exhibit spatial stickiness. As a result, it distorts the distribution of rents n and outside the zone, ultimately enhancing the ability of FTZs to capture these rents.

Methodologically, this study employs an integrated approach that combines total factor productivity (*TFP*) and geographic measures to investigate regional industrial agglomeration and neighbourhood effects.

1. First, *TFP* of listed firms is calculated following the methodology proposed by Levinsohn and Petrin [16] (henceforth *LP* model). This model takes into account firms’ input-output efficiencies.
2. Second, firm locations are fixed in estimation to facilitate a comparison between firms in and outside FTZs, allowing for an assessment of the influence of FTZ institutions.
3. Third, as some FTZs overlap with conventional economic zones, the distance of firms [5] to FTZs and/or conventional economic zones is considered to avoid double accounting.
4. Forth, time dummies are introduced to distinguish between the periods “before” and “after” the establishment of FTZs. Addressing endogeneity infirm data.
5. Finally, finite mixture models (*FMM*) are employed to identify varying industry responses to FTZ policies [9]. This helps determine the overall impact of today’s FTZs on regional growth–“prosper or beggar thy neighbour”.

The consequence of local government’s quasi*-*industrial policy is an uneven allocation of resources and a distortion in regional markets. Consequently, the concentration of certain industries in today’s FTZs is unlikely to generate positive neighbourhood effects for the peripheral areas.

The subsequent sections of this paper are organised as follows: Section 2 presents the data and variables used in the study; Section 3 outlines the empirical framework and presents the results; and Section 4 concludes the paper.

## 2. Variable estimation and data

### 2.1. Estimation of firm-level productivity

To assess a firm’s productivity, we employ the *LP* model. This model is adept at addressing the issue of synchronisation biases in the Solow residual [17], as well as overcoming the limitations associated with the Olley-Pakes method [18], which enforces a strictly monotonic relationship between inputs (proxies) and outputs.

In terms of changes in productivity (*σ*_*it*_) for firms maintaining Hicks neutrality [19], we set the log-transformed Cobb-Douglas function as follows:

$$y_{it}=\beta_{l}l_{it}+\beta_{k}k_{it}+\sigma_{it}+\varepsilon_{it}.$$ (1)

Incorporating considerations for endogeneity [20] and firms’ ownership attributes, we extend the *LP* method by introducing intermediate inputs *m*_*it*_ and the ownership of firms *γ*_*it*_. Thus, the expanded Cobb-Douglas function is estimated to derive expected output ${\hat{\delta }}_{it}$ and unobservable shock component *e*_*it*_ in the following manner:

$$y_{it}=\delta_{t}(l_{it},k_{it},\sigma_{it},\gamma_{it},m_{it})+e_{it}.$$ (2)

Accordingly, a firm’s productivity can be expressed as a function of coefficients *β*_*i*_,

$$\sigma_{it}(\beta )\equiv {\hat{\delta }}_{it}-\beta_{l}l_{it}-\beta_{k}k_{it}.$$ (3)

**Fig 1:**
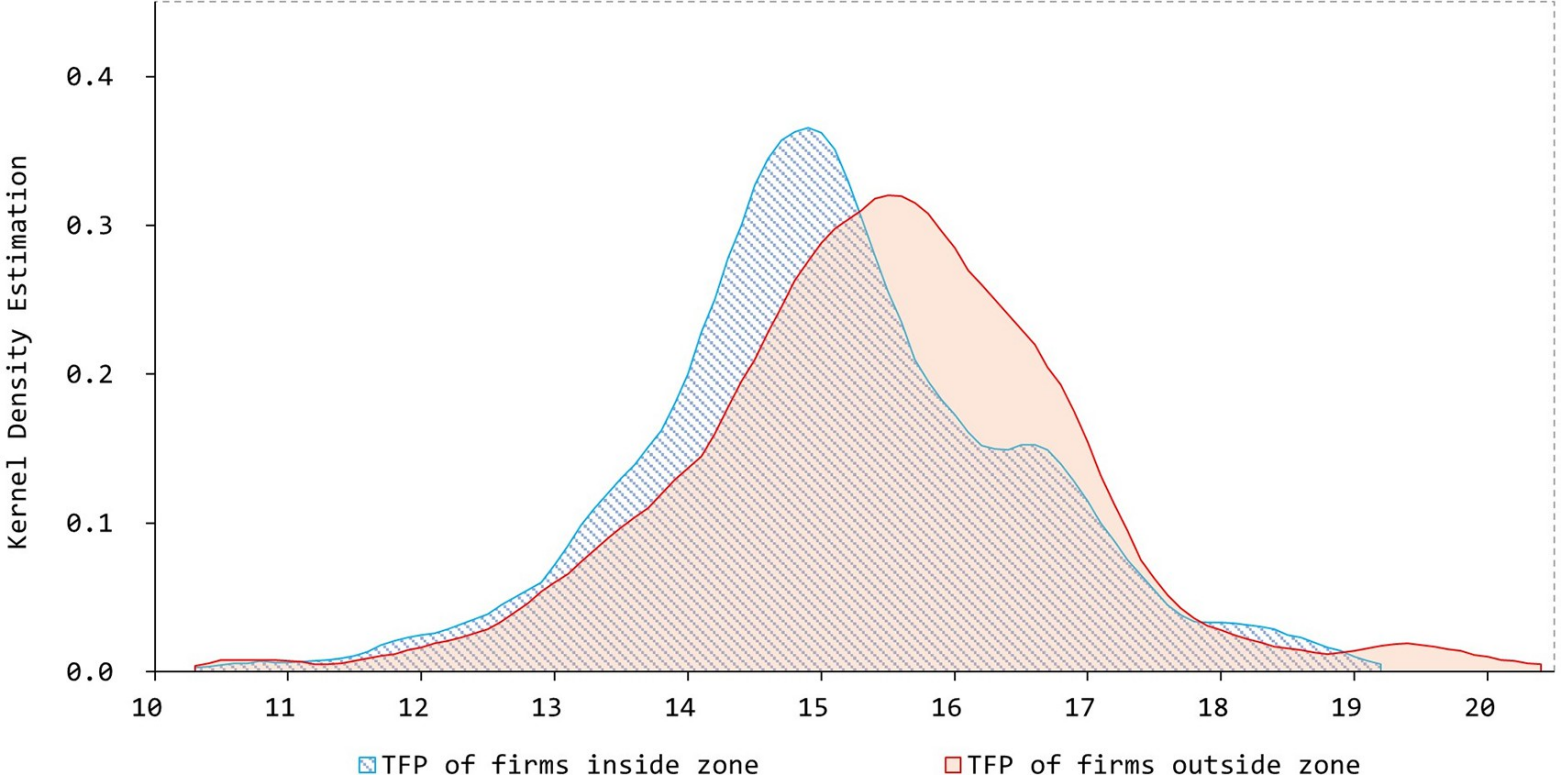
Kernel density estimation of *TFP*. Source: compiled by the authors.

Fig 1 illustrates the distribution of the estimated productivity denoted as $\hat{\sigma }$.

### 2.2. Other variables

A comprehensive set of corporate properties [5], alongside geographic, and macroeconomic control variables, has been incorporated to maintain analytical consistency. The dataset exclusively pertains to listed firms within Guangdong province, because it serves as the pilot region for the Chinese government’s FTZ trials.

Considering that Guangdong’s FTZ was initially established in 2014, our observation period is constrained to the years 2011–2017. This timeframe allows us to focus on the immediate response of firm productivity to FTZ policies and ensures a balanced pre- and post-test period for the quasi-experimental analysis. In addition to the previously calculated *TFP*, we employ variables such as cash payment to employees, payroll amount, the number of years a firm listed on the stock market, and the firm’s ownership structure to assess corporate attributes. We also integrate geographic information for each firm to examine the distance-related effects of industrial policy diffusion stemming from FTZs, comparing it with the conventional economic zones. To maintain analytical rigor, we control for the year and city of firm registration. Firm accounting data used in this study have been sourced from the China Stock Market and Accounting Research Database [21] and sample surveys with a summary of the data provided in Table 1.

**Table 1:** Summary statistics.

Variables	Obs.	Mean	S.D.	Min.	Max.	Description
	(1)	(2)	(3)	(4)	(5)	(6)
Corporate properties						
TFP of firms	2205	15.40	1.44	10.37	20.46	Total factor productivity estimated by *LP* method based on Eq (3).
Cash paid to employees	2205	19.12	1.41	14.91	24.78	Cash renumerations to all employees.
Payroll amount	2205	17.07	1.864	7.642	24.296	Various payments that enterprises offer to employees.
Number of years firm has been listed	2205	20.45	6.293	4.00	39.00	The number of years that the enterprise has been listed on the stock market.
Ownership property	2205	0.62	0.49	0.00	1.00	If the enterprise is privately owned take 1, or take 0.
Geographic location						
Firm located in free trade zones	2205	0.24	0.43	0.00	1.00	If the firm locates in a FTZ take 1, or take 0.
Located in economic zones	2205	0.18	0.39	0.00	1.00	If the firm locates in a conventional economic zone take 1, or take 0.
Distance to free trade zones	2205	55.63	91.15	0.00	346.10	Distance between the firm and the nearest FTZ.
Distance to the nearest economic zones	2205	7.08	6.73	0.00	50.00	Distance between the firm and the nearest conventional economic zone.
Number of economic zones	2205	6.43	2.85	0.00	13.00	The number of economic zones encompassing the city where the firm is registered.
Macro controls						
Year	2205	--	--	--	--	The year that observation value belongs to.
City of registration	2205	--	--	--	--	The city in which the observation firm is registered.

Source: compiled by the authors.

## 3. Empirical design and analysis

### 3.1. Firm locations and productivity spillovers

Our analysis began with a basic Eq (4), which serves as the core for investigating the presence of productivity spillovers emanating from FTZs:

$${productivity}_{it}=c+\alpha_{i1}{location}_{it}+\alpha_{i2}{property}_{it}+\sum \vartheta_{i}{city}_{it}+\sum \varphi_{i}{year}_{it}+\varepsilon_{it}.$$ (4)

In Eq (4), the term *location*_*it*_ encompasses the geographic information pertaining to the observed firms, while *property*_*it*_ includes all corporate properties. Additionally, *city*_*it*_ and *year*_*it*_ are used to respectively control for the city and the year of observation. The results of this analysis are presented in Table 2.

**Table 2:** Productivity spillovers from free trade zone.

	*TFP*							
	(1)		(2)		(3)		(4)	
Firm in free trade zone (Yes = 1)	–0.335	***	–0.308	***	–0.101		–0.104	
	(0.078	)	(0.083	)	(0.073	)	(0.072	)
Distance to the nearest zone centre			0.001		0.001	*	0.001	**
			(0.001	)	(0.000	)	(0.000	)
Cash paid to employees					0.556	***	0.310	***
					(0.024	)	(0.041	)
Payroll amount							0.222	***
							(0.031	)
Ownership property (Private = 1)	–0.609	***	–0.614	***	–0.135	**	–0.080	
	(0.072	)	(0.072	)	(0.067	)	(0.066	)
Number of years listed	0.028	***	0.028	***	0.026	***	0.023	***
	(0.005	)	(0.005	)	(0.005	)	(0.005	)
City of registration	–0.016	**	–0.024	**	0.006		0.001	
	(0.008	)	(0.012	)	(0.010	)	(0.010	)
Year	0.076	***	0.076	***	–0.017		–0.027	*
	(0.016	)	(0.016	)	(0.014	)	(0.014	)
Constant	–137.700	***	–138.100	***	38.720		58.630	**
	(31.830	)	(31.830	)	(28.920	)	(28.660	)
No. of observations	1818		1818		1818		1801	
R-squared	0.106		0.106		0.313		0.334	

Notes: Standard errors are reported in parentheses; ***p<0.01, **p<0.05, *p<0.1.

The negative coefficients observed for firms within the FTZs in columns 1 and 2 (–0.335 and –0.308 respectively) are statistically significant at the 99% confidence level, indicating a general lower productivity level for firms located in the FTZs. In columns 3 and 4, when distance to the FTZ, cash payment to employees, and payroll payable are introduced as additional factors, the direction of coefficients remains unchanged for both firms in and outside the FTZs. However, the statistical significance changes: the presence of the FTZ institution is no longer statistically significant for productivity changes among firms within the zone; while for firms outside the FTZ, the farther they are from the FTZ, the higher their productivity tends to be. This suggests that firms experience increased productivity when situated close to conventional industrial centres but away from FTZs. Consequently, Table 2 does not provide empirical evidence to support the notion of a positive neighbourhood effect of FTZs on regional growth.

It is worth noting that listed firms often undergo changes in location and primary business activities over time. Therefore, the influence of FTZ institutions on these firms may not occur randomly at the time of change. To better capture the immediate response of firms to the benefits of FTZs, Table 3 follows the approach developed by Alder *et al*. [22], by incorporating three time dummies: the year before, the year of, and the year after firms join an FTZ. These three time dummies are applicable to both new entrants into existing FTZs and firms located within areas that would later become FTZs.

**Table 3:** Immediate productivity change by FTZ institutions.

	*TFP*							
	(1)		(2)		(3)		(4)	
One year prior to being in zone (YES = 1)	0.053		0.056		–0.165	*	–0.224	**
	(0.062	)	(0.062	)	(0.090	)	(0.090	)
The year of being in zone (YES = 1)	0.008		0.012		–0.272	**	–0.337	***
	(0.069	)	(0.069	)	(0.107	)	(0.106	)
One year after being in zone (YES = 1)	0.429	***	0.435	***	0.010		–0.111	
	(0.085	)	(0.086	)	(0.146	)	(0.145	)
Ownership property (Private = 1)			0.175		0.162		0.150	
			(0.157	)	(0.146	)	(0.138	)
Cash paid to employees					0.403	***	0.168	
					(0.111	)	(0.118	)
Payroll amount							0.259	***
							(0.062	)
Year-Fixed Effect	Yes		Yes		Yes		Yes	
Constant	15.310	***	15.200	***	7.710	***	7.857	***
	(0.043	)	(0.113	)	(2.097	)	(2.049	)
No. of observations	1,818		1,818		1,818		1,818	
R–squared	0.059		0.059		0.085		0.114	

Notes: Standard errors are reported in parentheses

***p<0.01

**p<0.05

*p<0.1.

As is shown, the coefficients associated with the “one year prior to” and “the year of” a firm’s entry into the FTZ have shifted from being positive (as seen in columns 1–2) to becoming negatively significant (in columns 3–4). Conversely, the coefficients related to the “one year after” joining the FTZ have shown an opposite trend. In practical terms, firms that have recently relocated to or newly registered in FTZs often benefit from favourable zone policies, resulting in an immediate improvement in their short-term accounting performance.

It appears that Table 3 corroborates the findings presented in Table 2, leading to the conclusion that positive productivity spillovers resulting from FTZs have not yet fully materialized.

### 3.2. Technical discrepancies and industry clustering

An implicit assumption made in the preceding analysis was that the influence of zone institutions on individual firms was identical. However, in practice, the impact of these institutions on output varies significantly across different industries, leading to divergent outcomes even when subjected to the same set of zone institutions. There is a possibility that FTZ institutions may elicit multi-directional responses from various industries, which could elucidate the limited positive observations across FTZs as a whole.

In light of this, we have opted for the *FMM* to examine the unobservable heterogeneity among firms:

$$p(j|X,TFP)=\pi_{j}+f_{j}(TFP|X,{ℵ}_{j}),$$ (5)

$$\sum_{k}\pi_{k}f_{k}+exp(\gamma_{k}+\rho \gamma_{k})$$

Here, *ρ* represents the parameter of the mixture model, *X* stands for the explanatory variables, *k* denotes the number of classes, *π*_*k*_ signifies the probability for the *k*th class, and ℵ_*k*_ represents the parameter specific to each class.

When we set *k* = 3, the Bayesian Information Criteria is minimised, indicating the presence of three distinct pathways through which FTZ institutions can influence firms’ productivity. Table 4 categorizes these firms into different groups based on these pathways.

**Table 4:** Class identification by finite mixture model.

	*TFP*					
	Class 1		Class 2		Class 3	
	(1)		(2)		(3)	
Firm in free trade zone (Yes = 1)	0.056		–0.658	***	1.095	***
	(0.203	)	(0.121	)	(0.154	)
Distance to the nearest zone	0.083		0.076	*	–0.550	***
	(0.076	)	(0.044	)	(0.060	)
Cash paid to employees	–0.215		0.083		1.081	***
	(0.150	)	(0.060	)	(0.057	)
Payroll amount	0.843	***	0.177	***	–0.229	***
	(0.113	)	(0.034	)	(0.038	)
Ownership property (Private = 1)	0.148		0.102		–0.822	***
	(0.144	)	(0.095	)	(0.126	)
Number of years listed	0.009		0.036	***	0.027	***
	(0.011	)	(0.007	)	(0.010	)
City of registration	0.005		0.005		–0.063	***
	(0.026	)	(0.017	)	(0.020	)
Year	–0.093	**	0.042	*	–0.033	
	(0.041	)	(0.023	)	(0.025	)
Constant	190.700	**	–73.520		64.210	
	(82.010	)	(45.930	)	(50.270	)
No. of observations	1,801		1,801		1,801	

Notes: Standard errors are reported in parentheses

***p<0.01

**p<0.05

*p<0.1.

In Class 3 of Table 4, we observe that the productivity of firms located within FTZs is generally three times (*e*^1.095^) higher than firms outside, signifying a significant productivity boost attributed to the presence of FTZ institutions. Conversely, an opposing scenario emerges for intra-zone firms in Class 2, where the productivity of firms inside FTZs is only half (1/*e*^0.658^) that of firms located outside. This discrepancy becomes apparent when we account for geographic variations through city of registration. It becomes evident that the disparities between Classes 2 and 3 stem from technical differences in industry clustering. Notably, as none of the covariates are industry-dependent, the industry heterogeneity presented in Table 4 elucidates the variations in outcomes across all three classes.

### 3.3. Prosper or beggar thy neighbour

Considering the inherent diversity in economic performance among industries, the growth experienced by FTZs may largely be a result of their capacity to attract specific industries, thus driving accelerated growth within the zone. Table 5 provides insights from the results presented in Table 4 by highlighting the concentration of specific industries in each of the three identified classes.

**Table 5:** Industry performance and clustering.

Industries	Class		R^2^
	(3)		(4)
Manufacturing	–0.522	***	0.391
	(0.123	)	
Real Estate	1.711	***	0.420
	(0.275	)	
Leasing & Business Services	1.837	***	0.385
	(0.495	)	
Water Conservancy, Environment & Public Facilities	1.948	***	0.391
	(0.467	)	
Agriculture, Forestry, Animal Husbandry and Fisheries	0.080		0.365
	(1.123	)	
Electricity, Heat, Gas & Water Production & Supply Industry	–0.816		0.366
	(0.794	)	
Construction	0.013		0.365
	(0.509	)	
Wholesale and Retail Trades	0.091		0.365
	(0.298	)	
Transport, Storage and Post	0.603		0.369
	(0.366	)	
Information Transmission, Software & Computer Services	–0.023		0.365
	(0.168	)	
Others	–1.947	***	0.386
	(0.511	)	

Source: National Bureau of Statistics of China [23]. Standard errors are reported in parentheses

***p<0.01

**p<0.05

*p<0.1.

In practice, FTZs appear to exert a notable inhibitory effect on the productivity of manufacturing industries (as indicated by a coefficient of –0.522 in column 3). Conversely, they stimulate growth in the real estate and leasing industries (with coefficients of 1.711 and 1.837 respectively). This phenomenon can be attributed to the growth in housing and related financial industries, particularly after 2008, where these sectors exhibited significantly higher growth rates compared to the broader industry. For instance, between 2009 and 2022, the housing sector’s growth rate reached an impressive 393.29%, approximately one-third higher than that of the industry as a whole. This surge further solidifies its industry concentration in FTZs.

Today’s FTZs have a propensity to attract firms from high-growth industries; however, this does not necessarily imply a direct causal impact on individual firm growth. Similar to the case of Guangdong, there is a noteworthy overrepresentation of real estate-related industries within FTZs. This concentration of non-productive industries may lead to a permanent alteration in the regional factor distribution, thereby affecting productivity disparities both within and outside the zones. Consequently, unlike previous experiences, it becomes apparent that the most crucial lesson from today’s FTZs and their impact on regional growth is that the intricate relationship between industry clustering inside the zones and productivity changes in the surrounding areas is not always a “win-win” scenario.

## 4. Conclusion

There is a widely held belief that the concentration of industries plays a pivotal role in stimulating regional growth, making economic zones a key strategy adopted by local officials in their “promotion competition”. Present-day FTZs have evolved from the foundation laid by special economic zones. Possessing the utmost economic autonomy, local governments are dedicated to promoting and streamlining investments in industries that yield higher GDP output and/or generate greater economic rents.

In theory, firms that can benefit from the institutional framework of FTZs tend to prefer locating within these zones, where capital investments offer better returns, thus fostering faster growth in the zone area. However, in reality, the continuous reduction of transaction cost (i.e. tax and tariff reduction), deregulation, and financial incentives (i.e. quota and subsidies) have resulted in uneven industry concentration within FTZs.

From a broader perspective, basic economic theory suggests that factors of production become indifferent to agglomerating when institutional constraints are removed. In the context of spatial growth, the regional distribution of factor ratio can be permanently altered by industry clustering, leading to ongoing disparities in factor returns and productivity between zones and their surrounding areas. However, it is crucial to note that firms within FTZs are not necessarily more advanced in terms of productivity. If the policy-induced clustering of firms fails to enhance returns to inputs, it essentially amounts to a mere reallocation of productivity–a “siphon effect”.

When local governments focus their support on a single industry to drive GDP growth, they may inadvertently stifle potential innovations that other industries or locations could have promoted. The agglomeration resulting from favourable quasi-industrial policies may lead to the reallocation of productivity to FTZs or potentially result in the hollowing out of existing industries, ultimately having an adverse impact on regional growth–a “beggar-thy-neighbour” effect. In the long term, whether this effect is one of prosperity or detriment hinges on the interplay between the static loss stemming from resources reallocation and the dynamic gains associated with economies of scale during FTZ development.

## References

[1] Montinola G, Qian Y, Weingast BR. Federalism, Chinese Style: The Political Basis for Economic Success in China. *World Politics*. 1995; 48(1): 50–81. doi: 10.1353/wp.1995.0003

[2] Qian Y, Xu C. Why China’s Economic Reforms Differ: The M-Form Hierarchy and Entry/Expansion of the Non-State Sector. *Economics of Transition*. 1993; 1(2): 135–70. doi: 10.1111/j.1468-0351.1993.tb00077.x

[3] Zhou L. Jinsheng boyi zhong zhengfu guanyuan de jili yu hezuo–jianlun woguo difang baohu zhuyi he chongfu jianshe wenti changqi cunzai de yuanyin. *Jingji yanjiu*. 2004; 6: 33–40. Chinese.

[4] Aghion P, Cai J, Dewatripont M, Du L, Harrison A, Legros P. Industrial Policy and Competition. *American Economic Journal*: *Macroeconomics*. 2015; 7(4): 1–32. doi: 10.1257/mac.20120103

[5] Lin Y, Xiang W, Yu M. Quyu xing chanye zhengce yu qiye shengchanlv. *Jingji xue (jikan)*. 2018; 17(02):781–800. Chinese. doi: 10.13821/j.cnki.ceq.2018.01.14

[6] Crane B, Albrecht C, Duffin KM, Albrecht C. China’s Special Economic Zones: An Analysis of Policy to Reduce Regional Disparities. Regional Studies, *Regional Science*. 2018; 5(1): 98–107. doi: 10.1080/21681376.2018.1430612

[7] Siroën JM, Yücer A. Trade Performance of Free Trade Zones. The World Economy. 2017; 40(5): 1012–38.

[8] Ye P, Zhang H, Ma S, Yang F, Li Y. A Knowledge Map Study of an Application of a Smart Land Planning Free-Trade Zone and China’s Contribution. *Land*. 2022; 11(6):909. doi: 10.3390/land11060909

[9] Combes PP, Duranton G, Gobillon L. The Identification of Agglomeration Economies. *Journal of Economic Geography* 2011; 11(2): 253–66. doi: 10.1093/jeg/lbq038

[10] Krugman P. Increasing Returns, Monopolistic Competition, and International Trade. *Journal of International Economics*. 1979; 9: 469–79. doi: 10.1016/0022-1996(79)90017-5

[11] Krugman P. Increasing Returns and Economic Geography. *Journal of Political Economy*. 1991; 99(3): 483–99. doi: 10.1086/261763

[12] Criscuolo C, Martin R, Overman HG, Reenen JV. Some Causal Effects of An Industrial Policy. *American Economic Review*. 2019; 109(1): 48–85. doi: 10.1257/aer.20160034

[13] Jiang Y, Wang H, Liu Z. The Impact of the Free Trade Zone on Green Total Factor Productivity–Evidence from the Shanghai Pilot Free Trade Zone. *Energy Policy*. 2021; 148:112000. doi: 10.1016/j.enpol.2020.112000

[14] Fan G, Xie X, Chen J, Wan Z, Yu M, Shi J. Has China’s Free Trade Zone Policy Expedited Port Production and Development? *Marine Policy*. 2022; 137:104951. doi: 10.1016/j.marpol.2021.104951

[15] Li S, Liu J, Kong Y. Pilot Free Trade Zones and Chinese Port-Listed Companies Performance: An Empirical Research Based on Quasi-Natural Experiment. *Transport Policy*. 2021; 111:125–37. doi: 10.1016/j.tranpol.2021.07.022

[16] Levinsohn J, Petrin A. Estimating Production Functions Using Inputs to Control for Unobservables. *Review of Economic Studies*. 2003; 70(2): 317–41. doi: 10.1111/1467-937X.00246

[17] Solow RM. Heterogeneous Capital and Smooth Production Functions: An Experimental Study. *Econometrica* 1963; 31(4): 623–45. doi: 10.2307/1909163

[18] Olley GS, Pakes A. The Dynamics of Productivity in the Telecommunications Equipment Industry. *Econometrica* 1996; 64: 1263–97. doi: 10.2307/2171831

[19] Hicks J. *The Theory of Wages*. London: Macmillan; 1932. doi: 10.1007/978-1-349-00189-7

[20] Ackerberg DA, Caves K, Frazer G. Identification Properties of Recent Production Function Estimators. *Econometrica*. 2015; 83(6): 2411–51. doi: 10.3982/ECTA13408

[21] *China Stock Market and Accounting Research Database*. (n.d.) [cited 2022 June 15]. Available from. https://cn.gtadata.com Subscription required.

[22] Alder S, Shao L, Zilibotti F. Economic Reforms and Industrial Policy in A Panel of Chinese Cities. *Journal of Economic Growth*. 2016; 21(4): 305–49. doi: 10.1007/s10887-016-9131-x

[23] National Bureau of Statistics. *China Statistical Yearbook*. Beijing: China Statistical Press; 2010–2021 Editions.

